# Perceptions of people with Diabetes Mellitus after amputation: experiences and mental healthcare

**DOI:** 10.1590/0034-7167-2025-0226

**Published:** 2026-09-28

**Authors:** Bianca Brandão da Silva, Maria Giovana Borges Saidel, Maria Helena de Melo Lima

**Affiliations:** I Universidade Estadual de Campinas. Campinas, São Paulo, Brazil

**Keywords:** Diabetes *Mellitus*, Mental Health, Nursing Care, Mental Health Assistance, Qualitative Research., Diabetes *Mellitus*, Salud Mental, Atención de Enfermería, Atención a la Salud Mental, Investigación Cualitativa.

## Abstract

**Objectives:**

to understand, in light of Social Representation Theory, the perceptions of people with Diabetes *Mellitus* after amputation regarding mental healthcare, exploring the meanings attributed to these practices in a high-complexity hospital.

**Methods:**

a descriptive qualitative study conducted with ten participants undergoing outpatient follow-up after amputation. Data were collected through participant observation, semi-structured interviews, and field diary entries. Data analysis followed thematic content analysis.

**Results:**

three main thematic axes emerged: the invisible burden of diabetes; the ruptures and resignifications related to the disease; and the meanings attributed to mental healthcare.

**Final Considerations:**

the importance of the nursing team in mental healthcare is highlighted, being recognized as fundamental for the therapeutic relationship, health education, counseling and guidance, contributing to restoration of health from a holistic perspective.

## RESUMEN

**Objetivos:**

comprender, a la luz de la Teoría de las Representaciones Sociales, las percepciones de las personas con Diabetes *Mellitus* tras una amputación respecto a la atención en salud mental, explorando los significados atribuidos a estas prácticas en un hospital de alta complejidad.

**Métodos:**

estudio cualitativo descriptivo realizado con diez participantes en seguimiento ambulatorio tras una amputación. Los datos se recopilaron mediante observación participante, entrevistas semiestructuradas y anotaciones en diarios de campo. El análisis de datos se basó en un análisis de contenido temático.

**Resultados:**

surgieron tres ejes temáticos principales: la carga invisible de la diabetes; las rupturas y resignificaciones relacionadas con la enfermedad; y los significados atribuidos a la atención de la salud mental.

**Consideraciones Finales:**

se destaca la importancia del equipo de enfermería en la atención a la salud mental, siendo reconocido como fundamental la relación terapéutica, la educación en salud, el asesoramiento y la orientación, contribuyendo al restablecimiento de la salud desde una perspectiva holística.

## INTRODUCTION

It is estimated that the prevalence of Diabetes *Mellitus* (DM) will increase by 16% in the coming decades due to population aging. Currently, DM is a disease that occurs in both genders, being more prevalent in males. Brazil ranks sixth in the world, and is expected to increase from 16.6 million cases in 2024 to 24.0 million in 2050. Regarding healthcare costs, the United States leads with $404.5 billion, followed by China ($168.9 billion) and Brazil ($45.1 billion), highlighting the growing impact of DM as a public health priority^(1)^.

Studies show that the chronicity and complexity of living with DM can have a significant psychosocial impact^(2-6)^. DM is associated with a higher risk of developing mental health problems compared to the general population^(7,8)^. A systematic review indicated that depression and anxiety are associated with a higher risk of diabetes-related complications^(9)^.

Among the complications of DM, amputations of the lower limbs or parts of limbs generate physical and emotional limitations, resulting in psychological distress and stigmatization. Stigma, associated with loss of autonomy, intensifies the distress caused by the disease^(10)^. Furthermore, people with chronic conditions, including DM, face discrimination that can restrict their political, civil, and social rights^(11)^.

In this context, the importance of comprehensive care stands out, with an emphasis on mental healthcare, especially in the face of chronic conditions such as DM and its complications. Strategies for acceptance, therapeutic bonding, and emotional support are fundamental to dealing with psychological distress and promoting coping with vulnerabilities^(12)^. Mental healthcare refers to relational, communicative, and educational actions integrated into clinical care. These actions constitute a practice that supports autonomy, dignity, and coping mechanisms in the face of chronic illness and its complications^(13-15)^.

We adopted Social Representation Theory (SRT) as a framework, as it allows us to understand how shared meanings guide care practices. SRT articulates cognitive, affective, and cultural dimensions, guiding behaviors through anchoring (inscribing the new within the already known) and objectification (making the abstract concrete and communicable through images, metaphors, and examples). It considers the dynamics between the central core (stable elements that provide coherence) and the peripheral system (contextual and adaptive elements)^(16-19)^.

In the present study, SRT guided all stages: construction of an interview script on the meanings of the experience with DM, amputation, and mental healthcare; data collection attentive to language, metaphors, and contexts; thematic content analysis with emerging categories interpreted through anchoring-objectification and core-periphery relationship; and interpretation that articulates the representations to implications for mental healthcare in nursing.

Although DM and its complications are widely studied, there is a gap in qualitative research exploring, from the perspective of people experiencing amputations, the meanings attributed to the disease, amputation, and mental healthcare in hospital settings. This study seeks to understand how these individuals interpret and attribute meaning to such care, contributing to the improvement of more humanized and integrated practices.

## OBJECTIVES

To understand, in light of SRT, the perceptions of people with mental health issues after amputation regarding mental healthcare, exploring the meanings attributed to these practices in a high-complexity hospital.

## METHODS

### Ethical aspects

Considering the ethical issues involving human subjects, data were only collected after approval from the study field management and the *Universidade Estadual de Campinas* (UNICAMP) Research Ethics Committee. All participants signed the Informed Consent Form. To ensure anonymity, participants were identified by alphanumeric codes, and any information that could allow their identification was suppressed in the records and transcripts.

### Study design

This is a qualitative study of a descriptive-interpretative nature, appropriate for analyzing the meanings and intentions attributed to social practices^(19)^. The theoretical framework adopted was SRT, which guided the understanding of the phenomenon and the analysis of the narratives, allowing access to the symbolic and affective constructions about mental healthcare, as experienced by people with DM after amputation. To ensure the methodological rigor of this study, the COnsolidated criteria for REporting Qualitative research were used^(20)^.

### Study setting

The study was conducted in one outpatient unit and three inpatient units of a high-complexity hospital in Campinas, São Paulo, Brazil. The municipality, located in the southwestern region of the country, has approximately 1.2 million inhabitants and is divided into five health districts: North, South, East, Northwest, and Southwest^(21,22)^.

The hospital offers medium and high complexity care, meeting public health demands. In this context, the General Adult Outpatient Clinic, on Monday afternoons, welcomes people with DM and/or skin lesions. The multidisciplinary team, in collaboration with academic institutions, provides care. Nursing faculty conduct nursing consultations, which include guidance on the disease, promotion of self-care, assessment of the lower limbs, wound care, and guidance on DM management. In addition, they can be called upon for consultations in the hospital’s inpatient units.

### Study participants

Ten individuals with DM and amputation, treated at a high-complexity hospital, participated in the study. Of these, six were under outpatient follow-up and four were hospitalized in hospital units, also under outpatient follow-up. Only one person refused to participate. The sample was intentionally defined and closed by theoretical saturation, aiming to include subjects that reflected the particularities of the phenomenon studied^(23)^.

The inclusion criteria considered were age equal to or greater than 18 years, outpatient follow-up, diabetic foot condition, and a history of amputation of the limb or part of the limb as a consequence of DM complications.

### Data collection and organization

Data collection took place between March and October 2021, using two complementary techniques: participant observation and semi-structured individual interviews. During this period, systematic field diary entries were made documenting impressions, perceptions, reactions, and observed contexts. Participant observation provided data on the environment and interactions in their naturalness and complexity, allowing for the monitoring of nursing care activities and the relationships between the team and users. This observation was carried out in the outpatient clinic and inpatient units throughout the data collection period, with field diary entries recording impressions that subsequently enriched the analysis.

Individual interviews were conducted using a three-part structured instrument: (1) sociodemographic and clinical characteristics; (2) guiding questions, which included: “Tell me about what it is like to live with diabetes”; “Tell me about how you see yourself today”; “After the amputation(s), tell me about things that have changed in your daily routine”; “What feelings have you had or do you have about your life today”; “What do you think could help take care of your mental health here at the service?”; and “Tell me about the nursing team’s role in caring for your mental health”; and (3) observation during the interview of nonverbal aspects, such as posture, clothing, facial expressions, and body language. The interviews were conducted between April and October 2021, lasting an average of seven minutes, in locations convenient for participants and conducted by the same member of the research team.

All interviews were recorded, transcribed in full, and analyzed together with field diary entries, imported into NVivo 12 for management and coding, seeking to reconstruct experiences and perceptions about living with DM, self-perception, self-image, impacts of amputation, mental health practices, and the nursing team role in this care (exploratory axes of the structured instrument). To aid the analysis, a word cloud was created. Sample characterization data, due to their influence on mental health, were analyzed together with the interview content. SRT guided the interpretation of results, providing theoretical depth to the analytical process.

### Data analysis

After transcribing, organizing, and coding the data, a thematic content analysis was performed for later discussion from the perspective of SRT. This technique involves four stages: 1) pre-analysis; 2) material exploration; 3) processing of the results obtained; and 4) interpretation^(24,25)^. The categories emerged from the reading and coding of empirical material, in a collaborative and iterative process among three researchers, until a consensus was reached. The identification of units of meaning enabled the construction of thematic areas that were subsequently validated by the researchers. SRT allowed us to understand how the structures around the experience with DM, amputation, and mental healthcare are organized.

## RESULTS

Ten people with a diagnosis of DM and a history of amputation participated in the study. Their ages ranged from 53 to 66 years. Regarding ethnicity, five self-identified as white, three as mixed-race, and two as black. In terms of religion, four identified as Catholic, three as Evangelical, and one as Spiritist. Concerning education, four had completed high school; two had incomplete high school education; and four had not completed elementary school. Eight declared themselves married; one was single; and one was divorced. The majority were in a situation of labor vulnerability, with eight unemployed and two retired, seven of whom were on leave due to amputation.

Based on the narratives analyzed using SRT, meanings emerged regarding the experience with DM, the experience of amputation, and mental healthcare. These meanings were organized into three major thematic categories, as shown in Chart 1. In this chart, the phrases identified as “contents” correspond to condensed units of meaning.

**Chart 1:** Emerging thematic categories and content, Campinas, São Paulo, Brazil, 2024

Emerging thematic categories	Content (condensed units of meaning)
Experiences related to Diabetes Mellitus: the invisible burden	The disease that acts in silence; Guilt and regret; The routine that prevents care.
Amputation is not just cutting: ruptures and re-significations	The body I no longer recognize; Life on hold: work, home, identity; The symbolic power of spirituality and family.
Meanings attributed to mental healthcare: caring also means listening and affection	The word that sustains; A comforting presence; The gesture that disenchants; When one does not speak up, they do not receive support.

These empirical excerpts presented in the units of meaning reflect anchoring in everyday knowledge (work, faith, distress, care) and objectification of abstract experiences into concrete images (such as “weight” or “silence”) (Table 1), in line with SRT.

Shared perceptions about living with DM, changes resulting from amputation, and meanings attributed to mental healthcare contributed to the formation of the word cloud represented in Figure 1, revealing the most recurrent terms and the central elements of the social representations shared by participants.

**Figure 1:**
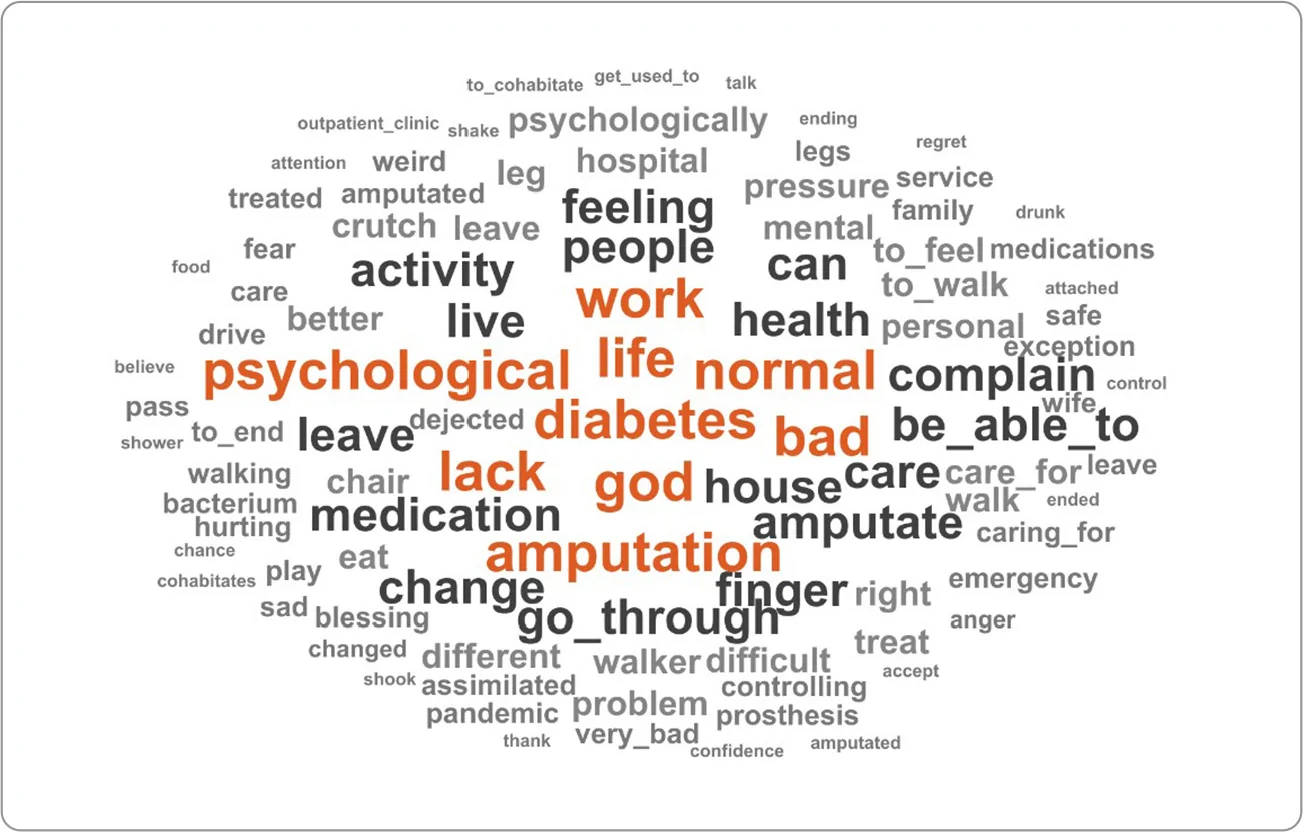
Word cloud generated in NVivo 12 from responses in individual interviews, Campinas, São Paulo, Brazil, 2024

Figure 1 was developed in conjunction with lexical analysis in NVivo 12. This tool highlighted recurring terms such as “diabetes”, “life”, “work”, “psychological”, “amputation”, “lack”, and “God”, which express central anchoring in participants’ social representations, reinforcing the relationship between illness, amputation, and mental health.

### Experiences related to Diabetes *Mellitus:* the invisible burden

In this category, representations of DM emerge as a silent, insidious, and guilt-inducing disease. Participants described the illness as a solitary experience, with little social visibility and often neglected until serious complications arise. The disease appears as an invisible agent that undermines autonomy, imposes restrictions, and carries a sense of personal failure, as revealed in the following excerpts:

*Terrible* [very blunt]*! Awful! You can’t eat anything; you can’t drink anything.* (E5)

*Ah, diabetes is a nasty disease that hurts and doesn’t heal* [...] *it’s bad because I have children to raise and I end up being a burden to them* [...] *sometimes my blood sugar drops, I think I’m sleeping, but I’m actually unconscious* [...] *I was arrested once because they thought I was drunk; then they found out it was diabetes.* (E6)

*Look, because it’s an illness where you don’t feel anything, you know, it acts silently, like, I’m normal, but one thing I’ve noticed is that when you don’t have it under control, you feel like you have the flu, you get tired, you know? You feel unwell, things like that.* (E8)

Challenges in developing self-care practices in daily life have been identified as factors that facilitate the emergence of complications, intensified by the exhausting routines of work settings.

*It’s quite complicated* [...] *if I could go back in time, I would be the king of physical activity today* [...] *I developed diabetes from not exercising. I spent 16 years working sitting down. I didn’t know the consequences* [...] *so, let’s get moving, do some physical activity, because it’s important to avoid developing diabetes.* (E2)

*Ah, it’s awful living with diabetes because we don’t have adequate services. At work, it’s hard work, it goes past the hours* [...] *that’s because I work in construction* [...] *when you have a lighter service, it’s easier* [...] *easier to control.* (E10)

The experience of living with DM was indirectly associated with psychological distress, anchored in feelings of restriction, guilt, and loss of control over one’s own life. These feelings reveal that the disease is perceived as a clinical condition marked by limitation, vigilance, and emotional strain.

### Amputation is not just cutting: ruptures and re-significations

Amputation emerged as a symbolic landmark of rupture in participants’ lives, promoting profound physical, emotional, and social transformations. The representations associated with the loss of a body part refer to the end of a cycle, the symbolic death of a previous identity, and the challenge of constructing a new self-narrative. Subsequent statements present feelings of frustration, embarrassment, sadness, a sense of emptiness, and/or fear. These experiences were associated with loss of autonomy, absence from work, and disruption of daily life, aspects that directly impact mental health.

*I’m thinner* [...] *I miss my amputated toe. It feels like there’s a void* [...] *it’s embarrassing, sad* [...] *my mind is fading, maybe because of the medication, but I’m more forgetful, without energy, without the vision I had before. Before I knew what to do, now I can’t discern. It feels like everything is fading, as if I’m at the end of the line* [...] *lost in the illusion of wanting to progress in life.* (E1)

*Today I almost fell while getting up with my walker. I need to use crutches and I’ve already managed to get a wheelchair for showering. It changes everything in life. I can’t work* [...] *I had a two-story house that I now need to pay someone to finish* [...] *my life isn’t over, but sometimes God gives you a push to get by when you’re complacent* [...] *a lot changes, the feeling is different, the neighbors look at you differently* [...] *everything is different.* (E6)

Alongside the losses and pain, some narratives point to processes of reinterpreting the experience, especially mediated by spirituality or family ties. These elements acted as coping mechanisms, offering meaning and support in the face of the rupture.

*So, I think this amputation was actually a reset, because, for some time now, I hadn’t been taking care of myself.* [...] *I have two children. I have my wife and, in a way, they depend on me* [...] *I had an amputation, but I didn’t die* [...] *you have to accept the situation and do everything, absolutely everything, to live the best way possible.* [...] *I’ve already learned to live with it.* (E8)

[...] *I am very religious and, in a way, this didn’t shake me much* [...] *I believe that everything is part of God’s purpose; nothing is by chance. He wants to teach me not to cling to things, because He could have taken my whole leg* [...] *or my life* [...] *I managed to assimilate it well; I feel that I am part of a greater purpose and that God is in control. I have never lacked anything at home, I have been resting and He has provided everything* [...] *if it weren’t in His plans, I would be suffering.* (E8)

Amputation also implied radical changes in lifestyle and work routine, highlighting the absence from professional activities as a direct consequence, in addition to causing significant emotional implications:

*I could be working. I stopped working because of that.* (E5)

[...] *I miss having a little foot* [on my side] [...] *it’s a radical change* [...] *I’m still away from work; I’m mostly at home taking care of myself* [...] *I hope to be free of the crutch and resume my normal life.* (E8)

[...] *I work independently. Right now, I can’t go up on the scaffolding or down on the scaffolding. I can’t do that right now, so I’m bringing people up there to do it for me.* (E9)

These representations reveal that amputation is experienced in an ambivalent way: it intensifies psychological distress and loss of identity, but it can also be reinterpreted as a new beginning, mediated by faith, hope, and family support. It is an experience that transcends the physical dimension, profoundly impacting participants’ mental health.

### Meanings attributed to mental healthcare: caring also means listening and affection

In this central category, social representations of mental healthcare emerge, often expressed indirectly, but intensely associated with the presence, connection, listening, and affection offered by professionals, especially the nursing team. Mental healthcare is represented by gestures, words, and presences that provide acceptance, encouragement, security, and confidence.

*Yes, here! Here I felt safe. And everyone treated me very well, I felt better here than at home, I don’t know why, but here I only receive kind words that encourage us. But there it’s just me and my wife, you know* [laughs]. (E1)

*Ah, they come and cheer us up so much and take care of us* [...] *we feel comforted.* (E5)

[...] *just look at how they treat you, you know? It’s already to give you a boost.* (E6)

A therapeutic relationship was also perceived as a form of careful correction. Field diary entries and participant observation evidenced the dimension of attentive and affectionate care in the daily interactions of outpatient practices, especially during moments of guidance on general care related to DM, providing guidance without punishment and reinforcing the feeling of support.

*When we make mistakes, they provide daily corrections* [...] *they correct us by pointing things out, “That’s not right”* [...] *they keep me on track.* (E2)

However, the nursing staff may have negative perceptions of care when comments from professionals evoke fear or feelings of helplessness.

[...] *I had just had my leg amputated, and she came to tell me that her father had contracted a bacterium, had his foot amputated, then his leg, and ended up passing away* [...] *but the nurse’s role is to encourage, not to create terror, and to help the patient maintain a positive mindset.* (E8)

The lack of acceptance in the face of suppressed emotional expressions was cited by one participant as a barrier to providing care.

*Don’t try to accommodate me because I haven’t expressed it to them* [...] *so I’m keeping it to myself.* (E3)

This individual perception aligns with the field diary entries, which document challenges in the nursing team’s work processes. In this particular unit, an overload of administrative tasks was observed in the outpatient clinic, accompanied by a reduction in activity in listening and support spaces. Furthermore, organizational failures were identified in the hospital, such as delays in the provision of care by the multidisciplinary team, which ultimately negatively impacted participants’ daily lives.

*One day I left here at 5:15 p.m., but my transport had already left at 4:00 p.m. I called my city* [...] *and they only arrived at 7:00 p.m. I got home almost at 8:00 p.m. When I walked in, I said, “Wow, this hospital killed me today”* [...] *but I wasn’t angry because I was here. If you’re treated well and politely, that’s already perfect.* (E2)

These statements demonstrate that mental healthcare, beyond specific techniques, is based on the symbolic recognition of a person, the validation of psychological pain, and the ethical and affectionate presence of professionals. Emerging as a central dimension, this care is anchored in bonds, encouraging words, welcoming gestures, and affective presence, especially from the nursing team. However, negative experiences and organizational failures have proven capable of weakening it, revealing its relationship with the symbolic recognition of psychological distress.

## DISCUSSION

Understanding mental healthcare from the perspective of people with mental disorders after amputation revealed a complex network of meanings and affects, socially organized and shared among participants. Using SRT, it was possible to access the meanings attributed to living with a chronic condition, the symbolic rupture caused by amputation, and the forms of care perceived-or absent-in coping with psychological distress^(17,18,26)^. These findings were strengthened by the triangulation of techniques, as the convergence between interviews, participant observation, and field diary entries allowed for the capture of speech, gestures, and interactions, increasing the representations’ interpretative depth and credibility^(15,24)^.

The representations constructed around DM have revealed it as a silent, treacherous, and relentless disease. The illness has been described as an experience marked by unpredictability and feelings of guilt: a condition that restricts and weakens autonomy, often perceived as a result of negligence or lack of time for self-care^(4,10)^. These representations are anchored in daily life and work relationships, which hinder healthy practices and, at the same time, hold individuals responsible for their illness^(11,27,28)^. In this logic, mental distress emerges indirectly: the burden of constant self-control, the fear of complications, and the lack of support are internalized as personal failure, intensifying the risk of psychological distress. Illness appears as both a clinical condition and a symbolic experience of surveillance, loss of freedom, and the threat of a rupture with one’s own identity^(13,14,17,27,28)^.

Studies indicate that people with DM have a higher risk of developing mental disorders, such as depression, anxiety, and burnout related to DM^(4,12,29)^, reinforcing the importance of emotional care. Other serious complications permeate the journey of those living with DM. Based on triangulation of methods, a study indicates that the incidence of diabetic foot and amputations is higher among males compared to females^(13)^. Although this relationship was not the primary focus of the investigation and the sample has limitations, the findings suggest a problematization of the vulnerability of men with DM, especially those who experience psychological distress after the amputation process.

Amputation was portrayed as a turning point in participants’ experiences - a division between before and after, requiring a subjective reorganization in the face of loss. It is not merely the physical absence of a body part, but an event that profoundly impacts self-image, functionality, social life, work, and future plans^(30,31)^. In a context where work is perceived as a structuring element of identity and social inclusion, labor barriers, such as unemployment, the mismatch between remuneration offered and the effort required, and difficulties adapting to work settings, must be incorporated into the dimensions of comprehensive care. The body, which was once an instrument of autonomy, now demands adaptations, new strategies for coexistence, and a constant process of self-reinvention.

However, representations of amputation are not univocal. While it is associated with feelings of frustration, shame, sadness, and fear, it is also reinterpreted as an opportunity for a new beginning, a reorganization of priorities, an appreciation of life, and the rebuilding of bonds. Spirituality, family support, and hope play a fundamental role in this process of symbolic reconstruction^(31-33)^. These representations reveal that amputation is fraught with ambivalence and that its subjective processing is directly associated with how care-or the lack thereof-manifests itself along this path.

Representations of mental healthcare emerge strongly associated with listening, acceptance, and the construction of a caring presence capable of offering meaning and security. Participants recognize, in the encouraging words, the affectionate tone of voice, and the daily attention of professionals, a powerful therapeutic space that alleviates distress and promotes subjective reorganization^(13-15)^. In this case, care is not described in technical or biomedical terms, but represented as a relationship. Emotional security, trust, bonding, and even attentive and affectionate correction appear as forms of care that positively mark the hospital experience. These perceptions reiterate the importance of practices that value the individual and their narratives, legitimizing their pain and opening space for dialogue^(14,34)^.

On the other hand, negative representations of care were also identified, associated with professional statements that aroused fear or despair and the absence of support in the face of restrained emotional expressions. Silence, when not listened to, becomes a way of making psychological distress invisible. This data reinforces the need for training and awareness-raising among healthcare teams to recognize subjective distress, especially in highly complex contexts such as hospital services^(15,35)^.

These representations demonstrate that mental healthcare is not limited to the presence of specialized professionals, but also involves building trusting relationships, validating the experience of others, and symbolically offering a space for listening and ethical presence. The nursing team stands out as an important figure in promoting and implementing this care^(36,37)^. When this relationship is possible, participants feel “better here than at home”, demonstrating that the power of sensitive care lies in recognizing the individual beyond the illness, even amidst pain and loss.

### Study limitations

This study was conducted in a single high-complexity hospital, which may limit the applicability of the findings to other healthcare settings. Furthermore, the purposive sample, composed exclusively of men, reflects unique, socially and culturally situated experiences, preventing the generalization of results. The interviews, with an average duration of seven minutes due to participants’ clinical conditions and hospital settings, were brief, but this did not compromise the depth of the analysis, supported by triangulation with participant observation and field notes.

### Contributions to nursing, health, or public policy

The contributions of this study emphasize the importance of integrating mental health into the care of people with DM and serious complications, such as amputations. The analysis of social representations revealed that the meanings attributed to the disease, amputation, and care directly impact coping strategies, and can be perceived both as sources of distress and limitation and as opportunities for re-signification and reconstruction of identity. In this scenario, the relevance of therapeutic relationships based on acceptance, active listening, emotional support, and recognition of psychological distress is highlighted. When adopted by healthcare professionals, including the nursing team, these practices favor engagement in self-care, subjective reconfiguration, and adherence to treatment. For public health, the findings highlight the need for policies that promote the training of nurses in mental health, the strengthening of social support networks, and the implementation of comprehensive care strategies.

## FINAL CONSIDERATIONS

The findings of this study show that mental healthcare for people with DM and amputations is represented as an experience marked by listening, acceptance, presence, and recognition of psychological distress. This care is symbolically anchored in the relationships established with healthcare professionals, among whom the nursing team appears as an important figure, especially when sensitive and humanized practices are offered. Social representations reveal that care is perceived not only as a technical action, but as a bond, a word that encourages, and a presence that supports. Thus, this study reinforces the need to invest in training strategies aimed at expanding the emotional and relational competencies of all professionals who work in the care of people with chronic conditions and psychological distress.

## Data Availability

The research data are available in a repository: https://doi.org/10.25824/redu/IBNEYX.
