# Supplementary material for: Perceptions of people with Diabetes Mellitus after amputation: experiences and mental healthcare
Source: Rev Bras Enferm. 2026 Sep 28;79:e20250226. doi: 10.1590/0034-7167-2025-0226 (PMC13623298; doi:10.1590/0034-7167-2025-0226)
Supplement: Translation

# Translation: Percepções de pessoas com Diabetes *Mellitus* após amputação: vivências e cuidados em saúde mental

**DOI:** 10.1590/0034-7167-2025-0226pt

**Authors:** Bianca Brandão da Silva, Maria Giovana Borges Saidel, Maria Helena de Melo Lima

**Affiliations:** I Universidade Estadual de Campinas. Campinas, São Paulo, Brasil

**Keywords:** Diabetes *Mellitus*, Saúde Mental, Cuidados de Enfermagem, Assistência à Saúde Mental, Pesquisa Qualitativa.

## Abstract

**Objetivos:**

compreender, à luz da Teoria das Representações Sociais, as percepções de pessoas com Diabetes *Mellitus* após amputação sobre os cuidados em saúde mental, explorando os significados atribuídos a essas práticas em um hospital de alta complexidade.

**Métodos:**

estudo qualitativo descritivo realizado com dez participantes em acompanhamento ambulatorial após amputação. Dados foram coletados por observação participante, entrevistas semiestruturadas e registros em diário de campo. Análise de dados seguiu a técnica de análise temática de conteúdo.

**Resultados:**

emergiram três principais eixos temáticos: o peso invisível do diabetes; as rupturas e ressignificações relacionadas à doença; e os sentidos atribuídos ao cuidado em saúde mental.

**Considerações Finais:**

ressalta-se a importância da equipe de enfermagem no cuidado à saúde mental, sendo reconhecida como fundamental para a relação terapêutica, educação em saúde, aconselhamento e orientação, contribuindo para o restabelecimento da saúde a partir de uma perspectiva integral.

## INTRODUÇÃO

Estima-se que a prevalência de Diabetes *Mellitus* (DM) aumente 16% nas próximas décadas, devido ao envelhecimento populacional. Na atualidade, o DM é uma doença que ocorre em ambos os gêneros, sendo mais prevalente no gênero masculino. O Brasil ocupa a sexta posição no mundo, devendo passar de 16,6 milhões de casos, em 2024, para 24,0 milhões, em 2050. Quanto aos custos de saúde, os Estados Unidos da América lideram, com 404,5 bilhões de dólares, seguidos por China (168,9 bilhões) e Brasil (45,1 bilhões), evidenciando o impacto crescente do DM como prioridade em saúde pública^(1)^.

Estudos demonstram que a cronicidade e a complexidade de viver com DM podem ter um grande impacto psicossocial^(2-6)^. O DM está associado a um maior risco de desenvolver problemas de saúde mental em comparação com a população em geral^(7,8)^. Revisão sistemática indicou que a depressão e a ansiedade estão associadas a um risco maior de complicações relacionadas ao DM^(9)^.

Entre as complicações do DM, as amputações dos membros inferiores ou parte dos membros geram limitações físicas e emocionais, resultando em sofrimento psíquico e estigmatização. O estigma, associado à perda de autonomia, intensifica o sofrimento da doença^(10)^. Além disso, pessoas com condições crônicas, incluindo DM, enfrentam discriminação que pode restringir seus direitos políticos, civis e sociais^(11)^.

Nesse contexto, destaca-se a importância do cuidado integral, com ênfase nos cuidados em saúde mental, especialmente diante de condições crônicas como DM e suas complicações. Estratégias de acolhimento, vínculo terapêutico e suporte emocional são fundamentais para lidar com o sofrimento psíquico e promover o enfrentamento das vulnerabilidades^(12)^. Os cuidados em saúde mental se referem a ações relacionais, comunicativas e educativas integradas ao cuidado clínico. Essas ações configuram uma prática que sustenta a autonomia, a dignidade e o enfrentamento diante da doença crônica e suas complicações^(13-15)^.

Adotamos a Teoria das Representações Sociais (TRS) como referencial, pois ela permite compreender como sentidos compartilhados orientam práticas de cuidado. A TRS articula dimensões cognitivas, afetivas e culturais, guiando condutas por meio de ancoragem (inscrever o novo no já conhecido) e objetivação (tornar concreto e comunicável o abstrato por meio de imagens, metáforas e exemplos). Considera-se a dinâmica entre o núcleo central (elementos estáveis que conferem coerência) e o sistema periférico (elementos contextuais e adaptativos)^(16-19)^.

No presente estudo, a TRS orientou todas as etapas: a construção do roteiro de entrevistas sobre os significados da experiência com DM, amputação e cuidados em saúde mental; a coleta atenta a linguagem, metáforas e contextos; a análise temática de conteúdo com categorias emergentes interpretadas pela ancoragem-objetivação e relação núcleo-periferia; e a interpretação que articula as representações às implicações para o cuidado em saúde mental na enfermagem.

Embora o DM e suas complicações sejam amplamente estudados, existe uma lacuna nas investigações qualitativas que explorem, sob a perspectiva das pessoas que vivenciam amputações, os significados atribuídos à doença, à amputação e aos cuidados em saúde mental no contexto hospitalar. Este estudo busca compreender como esses sujeitos interpretam e atribuem significados a tais cuidados, contribuindo para o aprimoramento de práticas mais humanizadas e integradas.

## OBJETIVOS

Compreender, à luz da TRS, as percepções de pessoas com DM após amputação sobre os cuidados em saúde mental, explorando os significados atribuídos a essas práticas em um hospital de alta complexidade.

## MÉTODOS

### Aspectos éticos

Considerando as questões éticas que envolvem os seres humanos, os dados somente foram coletados após aprovação da gestão do campo de estudo e do Comitê de Ética em Pesquisa da Universidade Estadual de Campinas (UNICAMP). Todos os participantes assinaram o Termo de Consentimento Livre e Esclarecido. Para assegurar o anonimato, os participantes foram identificados por códigos alfanuméricos, e qualquer informação que pudesse permitir sua identificação foi suprimida nos registros e transcrições.

### Tipo de estudo

Trata-se de estudo qualitativo de natureza descritivo-interpretativa, apropriado para analisar significados e intencionalidades atribuídas às práticas sociais^(19)^. O referencial teórico adotado foi a TRS, que orientou a compreensão do fenômeno e a análise das narrativas, permitindo acessar as construções simbólicas e afetivas sobre os cuidados em saúde mental, conforme foi experienciado por pessoas com DM após amputação. Para garantir o rigor metodológico do estudo, foi utilizado o *COnsolidated criteria for REporting Qualitative research* ^(20)^.

### Cenário do estudo

O estudo foi realizado em uma unidade ambulatorial e três unidades de internação de um hospital de alta complexidade em Campinas, São Paulo, Brasil. O município, localizado na região Sudoeste do país, conta com cerca de 1,2 milhão de habitantes e está dividido em cinco distritos de saúde: Norte, Sul, Leste, Noroeste e Sudoeste^(21,22)^.

O hospital oferece assistência de média e alta complexidade, atendendo às demandas de saúde pública. Nesse contexto, o Ambulatório Geral de Adultos, às segundas-feiras à tarde, acolhe pessoas com DM e/ou lesões cutâneas. A equipe multiprofissional, em colaboração com instituições acadêmicas, realiza o atendimento. Os enfermeiros docentes conduzem consultas de enfermagem, que incluem orientações sobre a doença, promoção do autocuidado, avaliação dos membros inferiores, realização de curativos e orientações sobre cuidados com o DM. Além disso, podem ser acionados para consultas nas unidades de internação do próprio hospital.

### Participantes do estudo

Participaram do estudo dez pessoas com DM e amputação, atendidas em um hospital de alta complexidade. Dessas, seis estavam em acompanhamento ambulatorial e quatro estavam internadas em unidades hospitalares, também sob acompanhamento ambulatorial. Apenas uma pessoa recusou a participação. A amostra foi definida intencionalmente e fechada por saturação teórica, visando incluir sujeitos que refletissem as particularidades do fenômeno estudado^(23)^.

Como critério de inclusão, consideraram-se idade igual ou superior a 18 anos, acompanhamento ambulatorial, condição de pé diabético e histórico de amputação do membro ou parte do membro em consequência da complicação do DM.

### Coleta e organização dos dados

A coleta de dados ocorreu entre março e outubro de 2021, utilizando duas técnicas complementares: observação participante e entrevistas individuais semiestruturadas. Durante esse período, foram realizados registros sistemáticos em diário de campo que documentaram impressões, percepções, reações e contextos observados. A observação participante forneceu dados sobre o ambiente e as interações em sua naturalidade e complexidade, possibilitando o acompanhamento das atividades assistenciais de enfermagem e das relações entre equipe e usuários. Essa observação foi realizada no ambulatório e nas unidades de internação ao longo da coleta, com registros em diário de campo das impressões que, posteriormente, enriqueceram a análise.

As entrevistas individuais foram conduzidas com um instrumento estruturado em três partes: (1) características sociodemográficas e clínicas; (2) questões norteadoras, que incluíram: “Fale-me sobre como é conviver com o diabetes”; “Conte-me sobre como você se vê hoje”; “Após a(s) amputação(ões), fale-me sobre coisas que mudaram na sua rotina de vida”; “Quais sentimentos você já teve ou tem em relação à sua vida hoje”; “O que você acha que poderia ajudar a cuidar da sua saúde mental aqui no serviço?”; e “Conte-me sobre a atuação da equipe de enfermagem no cuidado da sua saúde mental”; e (3) observação na entrevista de aspectos não verbais, como postura, vestimenta, expressões faciais e linguagem corporal. As entrevistas foram realizadas entre abril e outubro de 2021, com duração média de sete minutos, em locais convenientes para os participantes e conduzidas pelo mesmo membro da equipe de pesquisa.

Todas as entrevistas foram gravadas, transcritas na íntegra e analisadas em conjunto com os registros do diário de campo, importados para o *software* NVivo 12 para gerenciamento e codificação, buscando a reconstrução das experiências e percepções sobre o convívio com DM, autopercepção, autoimagem, impactos da amputação, práticas em saúde mental e o papel da equipe de enfermagem nesse cuidado (eixos exploratórios do instrumento estruturado). Para auxiliar a análise, foi elaborada uma nuvem de palavras. Os dados de caracterização da amostra, por influenciarem a saúde mental, foram analisados em conjunto com os conteúdos das entrevistas. A TRS orientou a interpretação dos resultados, conferindo densidade teórica ao processo analítico.

### Análise dos dados

Após a transcrição, organização e codificação dos dados, efetuou-se a análise temática de conteúdo para posterior discussão na perspectiva das TRS. Essa técnica prevê quatro etapas: 1) pré-análise; 2) exploração do material; 3) tratamento dos resultados obtidos; e 4) interpretação^(24,25)^. As categorias emergiram a partir da leitura e codificação do material empírico, em um processo colaborativo e iterativo entre três pesquisadoras, até se chegar a um consenso. A identificação de unidades de sentido possibilitou a construção de áreas temáticas que foram posteriormente validadas pelas pesquisadoras. A TRS permitiu compreender como se organizam as estruturas em torno da experiência com o DM, da amputação e dos cuidados em saúde mental.

## RESULTADOS

Participaram do estudo dez pessoas com diagnóstico de DM e histórico de amputação. As idades variam entre 53 e 66 anos. Referente à etnia, cinco se autodeclararam brancos, três, pardos, e dois, negros. Em relação à religião, quatro se identificaram como católicos, três, como evangélicos, e um, como espírita. Quanto à escolaridade, quatro concluíram o ensino médio; dois possuíam o ensino médio incompleto; e quatro não concluíram o ensino fundamental. Oito se declararam casados; um era solteiro; e um era divorciado. A maioria estava em situação de vulnerabilidade laboral, com oito desempregados e dois aposentados, sendo que sete estavam afastados devido à amputação.

A partir das narrativas analisadas à luz da TRS, emergiram sentidos atribuídos à experiência com o DM, à vivência da amputação e aos cuidados em saúde mental. Esses sentidos se organizaram em três grandes categorias temáticas, conforme o Quadro 1. Nesse quadro, as frases identificadas como “conteúdos” correspondem a unidades de sentido condensadas.

**Quadro 1:** Categorias temáticas emergentes e conteúdo, Campinas, São Paulo, Brasil, 2024

Categorias temáticas emergentes	Conteúdo (unidades de sentido condensadas)
Vivências em relação ao Diabetes *Mellitus:* o peso invisível	A doença que age no silêncio; A culpa e o arrependimento; A rotina que impede o cuidado.
Amputar não é só cortar: rupturas e ressignificações	O corpo que já não reconheço; A vida em suspenso: trabalho, casa, identidade; A força simbólica da espiritualidade e da família.
Sentidos atribuídos ao cuidado em saúde mental: cuidar também é escuta e afeto	A palavra que sustenta; A presença que conforta; O gesto que desencanta; Quando não se fala, não se acolhe.

Esses recortes empíricos apresentados nas unidades de sentido refletem ancoragens em saberes cotidianos (trabalho, fé, sofrimento, cuidado) e objetivação de experiências abstratas em imagens concretas (como “peso” ou “silêncio”) (Quadro 1), em consonância com a TRS.

As percepções compartilhadas sobre a convivência com o DM, as mudanças decorrentes da amputação e os sentidos atribuídos aos cuidados em saúde mental contribuíram para a formação da nuvem de palavras representada na Figura 1, revelando termos mais recorrentes e os elementos centrais das representações sociais compartilhadas pelos participantes.

**Figura 1:** Nuvem de palavras gerada no NVivo 12 a partir de respostas em entrevistas individuais, Campinas, São Paulo, Brasil, 2024

A Figura 1 foi elaborada em articulação com a análise lexical no NVivo 12. Esse recurso destacou termos recorrentes, como “diabetes”, “vida”, “trabalho”, “psicológico”, “amputação”, “falta” e “Deus”, que expressam ancoragens centrais das representações sociais dos participantes, reforçando a relação entre adoecimento, amputação e saúde mental.

### Vivências em relação ao Diabetes *Mellitus:* o peso invisível

Nesta categoria, emergem as representações do DM como uma doença silenciosa, insidiosa e geradora de culpa. Os participantes descreveram o adoecimento como uma experiência solitária, pouco visível socialmente e frequentemente negligenciada até o surgimento de complicações graves. A doença aparece como um agente invisível que mina a autonomia, impõe restrições e carrega um senso de fracasso pessoal, como revelam os trechos:

*Péssimo* [bem incisivo]*! Ruim! Não pode comer nada; não pode beber nada.* (E5)

*Ah, diabetes é uma doença ruim que machuca e não sara* [...] *é ruim porque tenho filho para criar e acabo dando trabalho pra eles* [...] *às vezes, a glicemia baixa, acho que estou dormindo, mas estou desmaiado* [...] *já fui preso porque acharam que eu estava alcoolizado; depois viram que era diabetes.* (E6)

*Olha, por ser uma doença que você não sente nada, né, é como fala, ela age no silêncio, né, assim, eu tô normal, mas só que uma coisa que eu percebi é que, quando você não tem ela controlada, você parece que tá com gripe, fica cansado, sabe? Com mal estar, essas coisas.* (E8)

Desafios para desenvolver práticas de autocuidado no cotidiano foram identificados como fatores que facilitam o surgimento de complicações, intensificados pelas rotinas exaustivas do ambiente de trabalho:

*É bem complicado* [...] *se eu pudesse voltar lá através, eu era hoje o rei da atividade física* [...] *eu adquirir a diabetes por não fazer atividade física. Fiquei 16 anos trabalhando sentado. Eu não sabia das consequências que dava* [...] *então, vamos se movimentar, fazer uma atividade física, porque tem a importância de não adquirir a diabetes.* (E2)

*Ah, é péssimo conviver com o diabetes, porque a gente não tem um serviço adequado. No trabalho, é um trabalho pesado, passa das horas* [...] *é que eu trabalho em construção de obra* [...] *quando você tem um serviço mais leve, é mais fácil* [...] *mais fácil de controlar.* (E10)

A vivência com o DM foi associada ao sofrimento psíquico de maneira indireta, ancorada em sentimentos de restrição, culpa e perda do controle sobre a própria vida. Esses sentidos revelam que a doença é percebida como condição clínica marcada por limitação, vigilância e desgaste emocional.

### Amputar não é só cortar: rupturas e ressignificações

A amputação emergiu como um marco simbólico de ruptura na vida dos participantes, promovendo profundas transformações físicas, emocionais e sociais. As representações associadas à perda de parte do corpo remetem ao fim de um ciclo, à morte simbólica de uma identidade anterior e ao desafio de construir uma nova narrativa de si. Os relatos subsequentes apresentam sentimentos de frustração, constrangimento, tristeza, sensação de vazio e/ou medo. Essas vivências foram associadas à perda da autonomia, ao afastamento do trabalho e à desorganização da vida cotidiana, aspectos que impactam diretamente a saúde mental.

*Estou mais magro* [...] *sinto falta do dedo do pé amputado. Parece que ficou um vazio* [...] *dá constrangimento, tristeza* [...] *minha cabeça está definhando, talvez pelos remédios, mas estou mais esquecido, sem ânimo, sem a visão que tinha antes. Antes sabia o que fazer, agora não sei discernir. Parece que tudo está se apagando, como se estivesse no fim da linha* [...] *perdido na ilusão de querer progredir na vida.* (E1)

*Hoje mesmo quase caí ao me levantar com o andador. Preciso usar muleta e já consegui uma cadeira de rodas para o banho. Muda tudo na vida. Não posso trabalhar* [...] *tinha uma casa de dois andares que agora preciso pagar alguém para terminar* [...] *minha vida não acabou, mas às vezes Deus dá um empurrão para a gente se virar, quando está acomodado* [...] *muda muita coisa, o sentimento é diferente, os vizinhos olham de outro jeito* [...] *tudo diferente.* (E6)

Ao lado das perdas e dores, algumas narrativas apontam para processos de ressignificação da experiência, especialmente mediados pela espiritualidade ou pelo vínculo familiar. Esses elementos atuaram como recursos de enfrentamento, oferecendo sentido e suporte diante da ruptura:

*Então, eu acho que essa amputação foi na verdade um reset, porque, de uns tempos pra cá, eu não estava cuidando.* [...] *eu tenho dois filhos. Tenho minha esposa e de uma certa forma eles dependem de mim* [...] *tive uma amputação, mas eu não morri* [...] *tem que aceitar a situação e fazer de tudo, de tudo, para viver da melhor forma.* [...] *já aprendi a conviver.* (E8)

[...] *sou muito religioso e, de certa forma, isso não me abalou muito* [...] *acredito que tudo faz parte de um propósito de Deus; nada é por acaso. Ele quer me ensinar a não me apegar às coisas, pois poderia ter tirado minha perna inteira* [...] *ou minha vida* [...] *consegui assimilar bem; sinto que faço parte de um propósito maior e que Deus está no controle. Nunca faltou nada em casa, tenho descansado e ele tem providenciado tudo* [...] *se não estivesse em seus planos, eu estaria sofrendo.* (E8)

A amputação também implicou mudanças radicais no estilo de vida e na rotina de trabalho, destacando o afastamento das atividades profissionais como uma consequência direta, além de acarretar implicações emocionais significativas:

*Que eu podia tá trabalhando. Parei de trabalhar por causa disso.* (E5)

[...] *faz falta um pezinho* [...] *é uma mudança radical* [...] *ainda estou afastado do trabalho, fico mais em casa me cuidando* [...] *espero me libertar da muleta e retomar minha vida normal.* (E8)

[...] *eu trabalho por conta. Agora não posso subir no andaime ou descer no andaime. Agora não posso fazer isso, e aí tô levando o pessoal lá pra fazer pra mim.* (E9)

Essas representações revelam que a amputação é vivida de modo ambivalente: intensifica o sofrimento psíquico e a perda de identidade, mas também pode ser ressignificada como um recomeço, mediada por fé, esperança e apoio familiar. Trata-se de experiência que atravessa a dimensão física, alcançando profundamente a saúde mental dos participantes.

### Sentidos atribuídos ao cuidado em saúde mental: cuidar também é escuta e afeto

Nesta categoria central, emergem as representações sociais sobre os cuidados em saúde mental, muitas vezes expressas de forma indireta, mas intensamente associadas à presença, ao vínculo, à escuta e ao afeto oferecido pelos profissionais, especialmente pela equipe de enfermagem. O cuidado em saúde mental é representado por gestos, palavras e presenças que proporcionam acolhimento, ânimo, segurança e confiança:

*Aqui sim! Aqui eu me senti seguro. E todos me trataram muito bem, eu me senti melhor aqui do que em casa, não sei porque aqui só recebo palavra bonita, que incentiva a gente. Mas lá é só eu e minha mulher, né* [risos]. (E1)

*Ah, eles vêm e animam a gente tanto e cuidam da gente* [...] *a gente se conforta.* (E5)

[...] *é só ver no jeito de tratar, entendeu? Já é pra te dar uma força.* (E6)

A relação terapêutica foi percebida também como uma forma de correção cuidadosa. Os registros em diário de campo e observação participante evidenciaram a dimensão do cuidado atento e afetivo nas interações cotidianas das práticas ambulatoriais, especialmente nos momentos de orientações sobre cuidados gerais relacionados ao DM, orientando sem punir e reforçando a sensação de apoio.

*Quando a gente está errando, eles têm a correção do dia-a-dia* [...] *eles corrigem a gente chamando atenção: “Isso não pode”* [...] *eles me colocam no eixo.* (E2)

No entanto, a equipe de enfermagem pode apresentar representações negativas do cuidado, quando falas de profissionais despertaram medo ou desamparo:

[...] *eu tinha acabado de amputar a perna e ela veio dizer que o pai dela pegou uma bactéria, amputou o pé, depois a perna, e acabou falecendo* [...] *mas o papel do enfermeiro é animar, não fazer terror, e ajudar o paciente a manter o pensamento positivo.* (E8)

A ausência de acolhimento diante de expressões emocionais contidas foi citada por um participante como uma barreira à oferta de cuidado:

*Não tenta acolher porque eu não expressei pra eles* [...] *então, eu tenho só pra mim.* (E3)

Essa percepção individual dialoga com os registros em diário de campo, que documentam desafios nos processos de trabalho da equipe de enfermagem. No referido núcleo, observou-se uma sobrecarga de tarefas administrativas no ambulatório, acompanhada de uma redução na atuação em espaços de escuta e acolhimento. Ademais, identificaram-se falhas organizacionais no hospital, como atrasos na prestação de cuidados pela equipe multiprofissional, que acabaram impactando negativamente a vida cotidiana dos participantes:

*Teve um dia que saí daqui às 17:15, mas meu transporte já tinha ido às 16:00. Liguei para a minha cidade* [...] *e só chegaram às 19:00. Cheguei em casa quase 20:00. Quando entrei, falei: “Nossa, hoje esse hospital me matou”* [...] *mas não fiquei com raiva porque estava aqui. Se você for bem atendido e educadamente, já é perfeito.* (E2)

Esses relatos evidenciam que o cuidado em saúde mental, para além de técnicas específicas, sustenta-se no reconhecimento simbólico da pessoa, na validação da dor psíquica e na presença ética e afetuosa dos profissionais. Emergindo como dimensão central, esse cuidado é ancorado em vínculos, palavras encorajadoras, gestos de acolhimento e presenças afetivas, sobretudo da equipe de enfermagem. Contudo, experiências negativas e falhas organizacionais se mostraram capazes de fragilizá-lo, revelando sua relação com o reconhecimento simbólico do sofrimento psíquico.

## DISCUSSÃO

A compreensão dos cuidados em saúde mental, sob a perspectiva de pessoas com DM após amputação, revelou uma complexa rede de sentidos e afetos, organizada socialmente e compartilhada entre os participantes. À luz da TRS, foi possível acessar os significados atribuídos ao viver com uma condição crônica, à ruptura simbólica causada pela amputação e às formas de cuidado percebidas - ou ausentes - no enfrentamento do sofrimento psíquico^(17,18,26)^. Esses achados foram fortalecidos pela triangulação de técnicas, pois a convergência entre entrevistas, observação participante e registros em diário de campo permitiu captar falas, gestos e interações, ampliando a densidade interpretativa e a credibilidade das representações^(15,24)^.

As representações construídas em torno do DM revelaram-no como uma doença silenciosa, traiçoeira e implacável. O adoecimento foi descrito como uma experiência marcada pela imprevisibilidade e pelo sentimento de culpa: uma condição que restringe e fragiliza a autonomia, muitas vezes percebida como resultado de descuidos ou falta de tempo para o autocuidado^(4,10)^. Essas representações se ancoram na vida cotidiana e nas relações laborais, que dificultam práticas saudáveis e, ao mesmo tempo, responsabilizam o indivíduo pelo adoecimento^(11,27,28)^. Nessa lógica, o sofrimento mental emerge de forma indireta: o peso do autocontrole constante, o medo das complicações e a ausência de suporte são internalizados como fracasso pessoal, intensificando o risco de sofrimento psíquico. A doença aparece como condição clínica e experiência simbólica de vigilância, perda de liberdade e ameaça de ruptura com a própria identidade^(13,14,17,27,28)^.

Estudos apontam que pessoas com DM têm maior risco de desenvolver transtornos mentais, como depressão, ansiedade e *burnout* relacionados ao DM^(4,12,29)^, reforçando a importância do cuidado emocional. Outras complicações graves permeiam o percurso de quem convive com o DM. A partir da triangulação de métodos, estudo indica que a incidência de pé diabético e amputações é superior entre indivíduos do gênero masculino em comparação àqueles do gênero feminino^(13)^. Embora essa relação não tenha constituído o foco primário da investigação e a amostra apresente limitações, os achados sugerem uma problematização sobre a vulnerabilidade de homens com DM, especialmente daqueles que vivenciam sofrimento psíquico após o processo de amputação.

A amputação foi representada como um marco nas vivências dos participantes - uma cisão entre o antes e o depois, exigindo uma reorganização subjetiva frente à perda. Não se trata apenas da ausência física de uma parte do corpo, mas de um evento que impacta profundamente a autoimagem, a funcionalidade, a vida social, o trabalho e os projetos futuros^(30,31)^. Em um contexto em que o trabalho é percebido como elemento estruturante da identidade e da inserção social, as barreiras laborais, como o desemprego, a inadequação entre remuneração oferecida e o esforço exigido, e as dificuldades de adaptação ao ambiente de trabalho devem ser incorporadas às dimensões do cuidado integral. O corpo, que antes era instrumento de autonomia, passa a demandar adaptações, novas estratégias de convivência e um constante processo de reinvenção de si.

Contudo, as representações da amputação não são unívocas. Se, por um lado, aparece associada a sentimentos de frustração, vergonha, tristeza e medo, por outro, também é ressignificada como uma oportunidade de recomeço, reorganização de prioridades, valorização da vida e reconstrução de vínculos. A espiritualidade, o apoio familiar e a esperança desempenham papel fundamental nesse processo de reconstrução simbólica^(31-33)^. Essas representações revelam que a amputação é repleta de ambivalências e que sua elaboração subjetiva está diretamente associada ao modo como o cuidado - ou a falta dele - se apresenta nesse percurso.

As representações do cuidado em saúde mental emergem fortemente associadas à escuta, ao acolhimento e à construção de uma presença cuidadora capaz de oferecer sentido e segurança. Os participantes reconhecem, nas palavras encorajadoras, no tom de voz afetuoso e na atenção cotidiana dos profissionais, um espaço terapêutico potente, que ameniza o sofrimento e promove reorganização subjetiva^(13-15)^. O cuidado, nesse caso, não é descrito em termos técnicos ou biomédicos, mas representado como relação. A segurança emocional, a confiança, o vínculo e até mesmo uma correção atenta e afetiva aparecem como formas de cuidado que marcam positivamente a experiência hospitalar. Essas percepções reiteram a importância de práticas que valorizem o sujeito e suas narrativas, legitimando sua dor e abrindo espaço para o diálogo^(14,34)^.

Por outro lado, também foram identificadas representações negativas do cuidado, associadas a falas profissionais que despertaram medo ou desespero e à ausência de acolhimento diante de expressões emocionais contidas. O silêncio, quando não escutado, torna-se uma forma de invisibilização do sofrimento psíquico. Esse dado reforça a necessidade de formação e sensibilização das equipes de saúde para o reconhecimento do sofrimento subjetivo, especialmente em contextos de alta complexidade como os serviços hospitalares^(15,35)^.

Essas representações demonstram que os cuidados em saúde mental não se limitam à presença de profissionais especializados, mas dizem respeito à construção de vínculos de confiança, à validação da experiência do outro e à oferta simbólica de um espaço de escuta e presença ética. A equipe de enfermagem se destaca como uma figura importante na promoção e efetivação desse cuidado^(36,37)^. Quando essa relação é possível, os participantes se sentem “melhor aqui do que em casa”, evidenciando que a potência do cuidado sensível está em reconhecer o sujeito para além da doença, mesmo em meio à dor e à perda.

### Limitações do estudo

Este estudo foi conduzido em um único hospital de alta complexidade, o que pode restringir a aplicabilidade dos achados a outros contextos de saúde. Além disso, a amostra intencional, composta exclusivamente por homens, reflete experiências únicas, situadas social e culturalmente, o que impede a generalização dos resultados. As entrevistas, com duração média de sete minutos devido às condições clínicas dos participantes e ao ambiente hospitalar, foram breves, mas isso não comprometeu a profundidade da análise, sustentada pela triangulação com observação participante e anotações em diário de campo.

### Contribuições para a Área

As contribuições deste estudo enfatizam a importância de integrar a saúde mental no cuidado de pessoas com DM e complicações graves, como amputações. A análise das representações sociais revelou que os significados atribuídos à doença, à amputação e ao cuidado impactam diretamente as estratégias de enfrentamento, podendo ser percebidos tanto como fontes de sofrimento e limitação quanto como oportunidades de ressignificação e reconstrução da identidade. Nesse cenário, destaca-se a relevância de relações terapêuticas baseadas em acolhimento, escuta ativa, suporte emocional e reconhecimento do sofrimento psíquico. Quando adotadas por profissionais de saúde, incluindo a equipe de enfermagem, essas práticas favorecem o engajamento no autocuidado, a reconfiguração subjetiva e a adesão ao tratamento. Para a saúde pública, os achados ressaltam a necessidade de políticas que promovam a capacitação de enfermeiros em saúde mental, o fortalecimento de redes de apoio social e a implementação de estratégias de cuidado integral.

## CONSIDERAÇÕES FINAIS

Os achados deste estudo evidenciam que os cuidados em saúde mental para pessoas com DM e amputações são representados como experiências marcadas pela escuta, acolhimento, presença e reconhecimento do sofrimento psíquico. Esses cuidados são simbolicamente ancorados nas relações estabelecidas com profissionais de saúde, entre os quais a equipe de enfermagem aparece como figura importante, especialmente quando práticas sensíveis e humanizadas são ofertadas. As representações sociais revelam que o cuidado é percebido não apenas como ação técnica, mas como vínculo, palavra que encoraja e presença que sustenta. Assim, este estudo reforça a necessidade de investir em estratégias formativas voltadas à ampliação das competências emocionais e relacionais de todos os profissionais que atuam no cuidado de pessoas com condições crônicas e sofrimento psíquico.

## Data Availability

Os dados de pesquisa estão disponíveis em repositório: https://doi.org/10.25824/redu/IBNEYX.
